# Medical Youth—Manhood—Age

**Published:** 1889-04

**Authors:** D. R. Wallace

**Affiliations:** Supt. East Texas Lunatic Asylum Terrell, Texas


DANIEL'S

### TEXAS MEDICAL JOURNAL.

A Representative Organ of the Medical Profession, and an Exponent of Rational Medicine; devoted to the Organization, Advancement and Elevation of the Profession in Texas.

PUBLISHED MONTHLY.—SUBSCRIPTION $2.00 A YEAR.

Vol. IV. AUSTIN, APRIL, 1889. No. 10.

### ORIGINAL ARTICLES.

CONTRIBUTED EXCLUSIVELY TO THIS JOURNAL.

*The Articles in this Department are accepted and published with the understanding that we are not responsible for, nor do we indorse the views and opinions of the writers by so doing.*

### MEDICAL YOUTH—MANHOOD—AGE.

BY D. R. WALLACE, M. D., LL. D.,

*Supt. East Texas Lunatic Asylum, Terrell, Texas.*

Read before the East Line Medical Society, and by vote of the Society contributed to DANIEL'S TEXAS MEDICAL JOURNAL for publication.

Fungar vice cotis, acutum reddere quæ ferrumvalet, exsors ipsa secandi.*

THE words of Bacon, "I hold every man a debtor to his profession from the which as men of course, do seek countenance and profit, so ought they of duty to endeavor themselves by way of amends to be a help and an ornament thereto"—written of the legal, hold with ten fold force when applied to the medical

*Epistola ad Pisones vs. 304-5. Q Horatii Flacci.

profession—it being the office of the former to help to execute, but of the latter equally to practice and help to build up; and will continue to be until in the words of Prof. Alfred L. Loomis, "there are new problems to be solved; no more wrongs to be righted; no faults to be corrected; no needs to be supplied; no new fields to be explored, where faithful workers may preform greater achievements than have been reached in the past".

MEDICAL YOUTH, naturally credulous, is inclined to trust in and to fallow authority. This tendency right enough within certain limits,—with the mental training and discipline, most enter upon their medical studies, is greatly intensified. Here a great mistake is often made:—the result, a good plowboy lost; a poor doctor made. But some young men are bright by nature, others stupid. To be sure, to the one, culture will prove a great help, to the other, a necessity. None so gifted but that in the prosecution of a medical career, he needs every possible help. Even a Drake or a Velpeau is immensely helped by preliminary training, and crippled and handicapped throughout his whole career by the want of it. Let the young man who taking such men for guides would enter upon the study of medical science without the needed mental discipline and intellectual outfit—his mind not having been cultivated into the varied and harmonious activities of which it is capable, using every available means of sharpening the perceptive powers, strengthening the judgment and adding precision and acuracy to the imagination, first settle the question with himself, whether he is quite sure he is by nature a Drake or a Velpeau.

Should no one, then, enter upon these studies but he who has passed successfully thro' a college or university curriculum? So far as the opinion of the author of this paper goes for anything —and it is the result of forty years of professional observation—he has no hestitation in stating:—it were better for all concerned, if this were the rule. That there are those who, not having enjoyed these advantages, are more useful than many of those who have, is nothing to the purpose—proves nothing; "quo ad" the general rule. General rules are not for exceptional cases.

It is not the contention that the candidate for matriculation into a medical college should be a perfect master of the Defferential or Integral Calculus, be able to acquit himself with credit in an examination upon the metaphysics of Berkely, Sir William Hamilton and Cousin, be learned enough in Greek prosody to appreciate the beauties of Euripides and Sophocles in the original; or even to read without a lexicon, Horace, Tacitus or Juvenal; but it is insisted as indispensable that he should have a disciplined mind, able to follow up long intricate processes of thought, and to elaborate from them his own conclusions.

And it may be added, the more of Mathematics, Greek, Latin and Metaphysics he has along with this *sine qua non* the better for himself and future patients. As preliminary to entering upon the study of the medical sciences, the education should include a minimum of doctrine and dogma, a maximum of the implements of intellectual research, together with any amount of that sort of mental discipline, enabling its possessor to deduce a conclusion from its premise, to estimate the value of evidence, and to form his own theory of things. The first opinion may be crude. It has been said, and cannot be too much and often insisted upon, that skeptical activity is better than dogmatic torpor.

Think—the truth, if may be, but *think*. In itself, a fact may be a very useless, stupid thing, becomes invested with interest and value only when regarded in its relation to other facts, as an illustration of some principle, or as an item of proof for, or against some theory.

THE MEDICAL TEACHING OF THE SCHOOLS, of the United States, it may be remarked, fully abreast with that of the best Institutions of Europe, is conducted in accordance with the very best scientific methods, and engaging the best intellect, has reached a perfection leaving little to be desired.

The most advanced, in fact, the nearest approach to perfect teaching in an age, characterized as this is in every department by unparalleled activity, it is believed, is to be found in our best Medical Institutions. Were a word of criticism in place, where there is so little to find fault with, so much to commend, it would find justification in the fact, certainly to be deprecated in theory, but in practice difficult of correction, viz.: in matriculating those whose mental outfit is such as to make their scientific method, and excellent instruction to such matriculants, *pearls before swine.*

THE DOCTOR AT WORK.

His medical studies completed, launched upon his career, the youth, manhood and age of the medical man bring with them corresponding obligations to himself and his fellows. These several stages have their respective adaptations, suited to special sorts of mental occupation growing out of experience and intellectual characteristics of time of life. It is not asserted, of course, that the labors of the different stages do not run into and overlap. In his intellectual growth, when allowed its orderly development, unchecked by untoward circumstances, not arrested by the solicitations of the passions, man's inclination follows adaptation. He likes best what he can do best. The lines of the great anatomist of human nature:

> "No profit grows where no pleasure is ta'en;
> In brief, sir, study what you most affect,"

finds apt illustration in the life work of the medical man.

THE YOUNG DOCTOR

glancing over the pages of a noted author, or listening to the lectnre of the renowned professor, accepts the teachings of the one, and the dicta of the other with a submissive credulity altogether unbecoming the vigor of manhood, or the maturity of age. These teachings, it is for the young doctor, during the first years of his professional life, when patients are few and leisure abundant, to examine and test. It is a misfortune for these years to be too much occupied with active practice, leaving little time for study and reflection.

PROFESSIONAL MANHOOD, is the time during which the doctor comes most directly in contact with professional life; with its varied duties and activities. He has brought to bear upon the opinions and theories of youth, the vigor and power of his mature faculties. The credulity of youth is out-grown, and replaced by *nullins addictus jurare in verba magestri*;—wont to swear in the words of no master. In the present state of medical knowledge, what passes as medical literature, is of course, in many cases, mere theory; there being even among the best informed, little consensus of opinion upon many subjects.

The gibe of Voltaire, that the art of the Doctor consists in pouring drugs, of which he knows nothing, into stomachs of which he knows less, is as untrue as it is cynical. Still there is truth in it. The mind confronts no obscurer problems than those found in disease, and its cure. Besides, the rapid advance of the healing art during the last half century, growing out of the unprecedented material development, has not given time to test; to select the true and reject the false. "Medicine," as remarked by another, "which once relied on tradition and authority, is now rapidly seeking for its basis the natural sciences," and hence the uncertainty in which it is not seldom invested. This is regarded by the thoughtless as an opprobrium, but seen in the light of the facts is rather a compliment. It is evidence of growth, and inchoate condition, of a transition period, through which it is passing from a lower to a higher state of development, from a system of empiricism based on authority, to one of scientific accuracy based upon induction, and a deeper insight into the laws of nature. It is within living memory that medical practice rested almost wholly upon empiricism—physiology and pathology, the main pillows of rational scientific medicine, having originated within the present century, at least in their relation to therapeutics. Such a mass of facts and principle has of late years been pouring in upon scientific medicine, there has not been time for them to take their proper places, to be assigned their relative position. No reproach, this, an augury of good rather, portending a grand future for rational medicine. Here then, is a grand work for professional manhood, worthy to engage its strength and vigor, to examine the facts and principle, and to test the theories based upon them.

Here it is that each can get in his work—each working for all, all for each. Let none underestimate it. The great temple of rational scientific medicine is to be builded *in these years*; but before even the foundation can be laid, the debris of authority and empiricism must be removed. Paraphrasing the words of England's great naval commander, upon an historic occasion, here it is the profession "expects every man to do his duty."

It is a sad—but no more sad than true—commentary upon human advancement that it should require more labor to displace error than to establish truth. Nearly twenty-five centuries passed away after Pythagoras had conceived the helio-centric, or true theory of the planetary system before it was accepted. Why? Because the mind of the race was preoccupied by error—the Ptolmaic conception had to be dislodged before the Copernican, or true theory could be received. "The inaudible, noiseless foot of time" brings

PROFESSIONAL AGE, and age, reflection. The ages of professional manhood duly improved, in age is wisdom. Old men for counsel, young men for war. The vigor of youth and manhood having been passed in warring upon hasty deductions and false theories, and in the pursuit of truth, the age of reflection takes charge of results.

The history of human progress furnishes not a few instructive facts. Copernicus, a universal genius, mathematician, theologian, physician, astronomer, the discoverer of the theory of the planetary system, commenced and pushed to an early completion his great work, "De Cœlestium Orbium Revolutionibus," destined to render his name immortal. Though urged by his friends and the learned all over Europe to publish the result of his labors, this tireless, patient worker kept on, year after year, touching, retouching and revising it for thirty years—his eyes now dimmed with years, having been gladdened with the sight of the first printed copy only a few days before they were closed in death.

It was reserved for the great discoverer of gravitation to verify the three empiric laws of Kepler, and to add the coup de grace to the theory of Copernicus. The immortal Newton conceived for the first time the law of gravitation in 1666, he published the "Principia," containing the demonstration in 1686. His great work on optics begun in 1675, was not permitted to see the light until 1704.

THE ORIGIN OF SPECIES BY MEANS OF NATURAL SELECTION, was published in 1859, when the author was fifty years old. It was commenced when he was twenty-two. Probably no book, certainly none in modern times, ever exerted so much influence in so short a time over human thought, ever produced so great a revolution in the literature of the race. It will give some idea of the power exerted by the labors of this incomparable genius to state that a mere catalogue of the literature of Darwinism entitled "Darwinische Theorie," by J. W. Sprengel of Berlin, gives thirty-six octavo pages to titles of books devoted to this subject, and contains the names of three hundred and twelve authors. History furnishes hundreds of similar instances, not so illustrious, but equally instructive. They are mentioned in this connection for the purpose of introducing the subject of medical literature; worse, cruder it is believed than that originating in any other department of thought.

To say that the country is flooded with it is simply to put in words what everybody knows;—to add that much of it is not worth the paper upon which it is written, is but stating what every thoughtful medical man knows, comes far short of the truth. It is not simply that it does no good,—it does immense harm. To the young and ill-taught, this popular trash is much more attractive than instructive matter. Perhaps it is not overstating the fact, to add that a large number of uncultivated medical men waste time enough over this poor stuff to make them if devoted to profitable study, useful in their profession. It lowers the standard of medical literature, it vitiates the taste of its readers. It is to be hoped it does some good, but what, if any, it would be hard to state. It would not be difficult to name medical journals of no mean circulations, in which, perhaps, the advertisements constitute the most profitable reading to be found in their colnmns;—a mere glance, being sufficient to show any one with the least acquaintance with the medical sciences, that the bulk of reading matter is made up of cases of not the least scientific value—of crude, immature opinions, and of the self-inflated conclusions of folly and ignorance.

PROFESSIONAL AGE, THE AGE OF REFLECTION, can occupy itself in no way more profitably for the advancement of rational scientific medicine—supposing of course the period of youth and vigor of manhood, passed as already indicated—than in putting in shape for instruction and preservation the real discoveries and practical results of times,—results as different from and as superior to the crass views and inflated nonsense of the thoughtless ignoramus desiring simply to see his name in print, as the diamond is different from, and superior to the crude coal.

It is little to be sure, that any one individual can contribute in a short lifetime. Let none be discouraged on this account.

If the many littles that make the much,—it is the quality not quantity. A lady friend of the writer, consulting the veteran botanist,—the late Doctor Asa Gray, of Harvard, in regard to the publication of a botany of the flora of Texas,—a work she had just prepared with great labor,—remarked: "Really, Doctor, I feel ashemed to submit to your inspection the result of my poor labors having been able to add so little to the general subject". "My dear," answered the great man," it is only a unit, the best of us can contribute after a lifetime of study to any subject; but of these units the material development of the nineteenth century is the aggregate result.

